# LncRNA FAM225B Regulates PDIA4-Mediated Ovarian Cancer Cell Invasion and Migration via Modulating Transcription Factor DDX17

**DOI:** 10.1155/2023/3970444

**Published:** 2023-09-07

**Authors:** Chanjiao Yao, Lingjuan Zeng, Qin Liu, Xiaoxin Qiu, Chunyan Chen

**Affiliations:** ^1^No. 2 Obstetrics and Gynecology Department, Hunan Provincial People's Hospital (The First Affiliated Hospital of Hunan Normal University), Changsha, China; ^2^Obstetrics and Gynecology Department, Hunan Provincial People's Hospital Xingsha Branch (People's Hospital of Changsha County), Changsha, China

## Abstract

**Objective:**

This study aimed to explore the roles and mechanisms of lncRNA FAM225B and PDIA4 in ovarian cancer.

**Methods:**

RT-qPCR and Western blot assays were performed to detect the expression levels of the lncRNAs FAM225B, DDX17, and PDIA4 in the serum of patients with ovarian cancer and cell lines. Cells were transfected with lncRNA FAM225B- and PDIA4-related vectors to determine the malignant phenotypes using functional experiments. The mutual binding of lncRNA FAM225B and DDX17 was verified using RNA pull-down and RIP assays.

**Results:**

The expression of lncRNAs FAM225B and PDIA4 was decreased in the serum of patients with ovarian cancer and cell lines. Restoration of lncRNA FAM225B or PDIA4 reduced cell proliferation, migration, and invasion abilities and elevated the apoptosis rate, whereas suppression of lncRNA FAM225B or PDIA4 exhibited an inverse trend. RNA pull-down and RIP assays revealed a direct interaction between lncRNA FAM225B and DDX17. ChIP assay revealed a relationship between DDX17 and the PDIA4 promoter. LncRNA FAM225B and DDX17 positively regulate PDIA4 expression. Downregulation of PDIA4 expression counteracts the suppressive effect of lncRNA FAM225B overexpression in ovarian cancer cells.

**Conclusion:**

This research study supports the fact that lncRNA FAM225B in ovarian cancer can upregulate PDIA4 by directly binding to DDX17, inhibiting the activities of ovarian cancer cells.

## 1. Introduction

Ovarian cancer is a heterogeneous gynecological malignancy with a complicated etiology and pathology [1]. The risk factors for ovarian cancer include genetic and environmental factors, such as geographic differences and family history [2, 3]. Chemotherapy is a commonly used therapeutic approach to ovarian cancer, but its efficiency is limited by drug resistance [4]. Early diagnosis is the most effective method to prevent advanced ovarian cancer [1]. Currently, improved technologies for predicting the risk of ovarian cancer are being used, and they take into account both genetic and epidemiological factors [5]. However, reliable diagnostic approaches and indicators for early screening of ovarian cancer are in their infancy; thus, identifying clinical biomarkers is critical for ovarian cancer treatment.

Long noncoding RNAs (lncRNAs) are capable of regulating diverse biological processes, including differentiation, development, and the cell cycle [6]. Evidence supports the implication of lncRNAs as either triggers or suppressors of cell viability, metastasis, and tumor development in ovarian cancer [7–10]. The lncRNA family with sequence similarity 225 members B (FAM225B, also called LINC00256B) is a newly discovered lncRNA whose expression is altered in several cancers, such as renal cell carcinoma, bladder cancer, and papillary thyroid carcinoma [11–13]. However, the role of FAM225B in ovarian cancer remains unclear. LncMAP predicted that lncRNA FAM225B would inhibit protein disulfide isomerase family member 4 (PDIA4) by binding to transcription factor DEAD (Asp-Glu-Ala-Asp) box helicase 17 (DDX17). Notably, PDIs play significant roles in cancer progression [14, 15], and PDIA4 might be a therapeutic marker for managing ovarian cancer [16]. Moreover, DDX17 functions as a transcriptional coactivator for diverse transcription factors [17, 18] to modulate tumorigenesis in malignancies, including breasts, colon, and lungs [19–21]. However, it is not apparent whether DDX17 regulates PDIA4 in ovarian cancer cells.

In this study, we aimed to determine the underlying mechanism of the lncRNAs FAM225B and PDIA4 in ovarian cancer development.

## 2. Materials and Methods

### 2.1. Ethics Statement

This study was approved by the local Ethics Committee of Hunan Provincial People's Hospital. Written informed consent was obtained from all patients.

### 2.2. Clinical Sample Collection

Serum samples (cancer group) were collected from March 2019 to December 2020 from 32 patients with ovarian cancer (aged 25–58 years; median age 41 years) and 10 healthy controls (HCs) (aged 25–48 years; median age 37 years) from Hunan Provincial People's Hospital. The samples that were collected were stored in liquid-nitrogen tanks. None of the patients in the cancer group had undergone radiotherapy or chemotherapy before surgery.

### 2.3. Cell Culture and Transfection

Human ovarian cancer cell lines (SKOV3, OVCAR-3, and OV90) and one human ovarian epithelial cell line (HOSE) purchased from the American Type Culture Collection (Rockville, MD, USA) were cultured at 37°C in RPMI-1640 (72400120, Gibco, New York, USA), containing 10% fetal bovine serum (FBS, 16140071, Gibco) with 5% CO_2_.

Overexpression FAM225B vector (OE-FAM225B), silenced FAM225B expression vector (sh-FAM225B), overexpression DDX17 vector (OE-DDX17), knockdown DDX17 expression vector (sh-DDX17), overexpression PDIA4 vector (OE-PDIA4), knockdown PDIA4 expression vector (sh-PDIA4), negative control of overexpression vector (OE-NC), and negative control of knockdown vector (sh-NC) were obtained from GeneChem (Shanghai, China). One day before cell transfection, the cells were seeded at 3 × 10^5^ cells/dish onto a 60 mm Petri dish and cultured for 24 h. The vector (3 *μ*g) was incubated with Lipofectamine 2000 reagent (11668019, Invitrogen, California, USA) and Opti-MEM I Reduced-Serum Medium (31985062, Gibco), followed by the addition of 8 ng/mL Polybrene (TR-1003, Sigma-Aldrich, St. Louis, MO, USA). The cells were incubated for 48 h in the medium containing the vector.

### 2.4. Colony Formation Assay

SKOV3 and OVCAR-3 cells in the logarithmic growth phase were detached using 0.25% trypsin, triturated into single cells, and suspended in culture medium containing 10% FBS for later use. The cell suspension was seeded onto a 6-well plate containing 10 mL of prewarmed culture medium (37°C) at a density of 500 cells per dish and gently shaken to disperse the cells evenly. The cells were incubated for 2-3 weeks at 37°C and 5% saturated humidity. When visible colonies were observed on the Petri dishes, the culture was terminated. After removing the supernatant, the remaining cells were washed carefully with phosphate-buffered saline (PBS) twice and fixed for 15 min in 5 mL of 1 : 3 acetic acid/methanol. After the fixative solution was removed, the staining process was performed for 1,030 min with an appropriate amount of Giemsa dyeing solution, and the dyeing solution was gradually rinsed with running water and air-dried. Subsequently, the plate was inverted and overlaid with a transparent mesh film. The colonies were directly counted with the naked eye, or the number of colonies with more than 10 cells was counted using a microscope (low-power lens).

### 2.5. Wound-Healing Assay

The cells were treated for 4 h with 100 *μ*g/mL mitamycin C (HY-13316, MCE, US) and then seeded onto 12-well plates at a density of 1 × 10^5^. When the cells reached 100% confluence, a scratch was scraped with a 10 *μ*L pipette tip vertically at the bottom of the well. The detached cells were removed by rinsing thrice with Dulbecco's PBS (DPBS, 14190250, Gibco). A fresh medium containing 2% FBS was added. Cells in the same vision at hours 0 and 24 were imaged using an Olympus inverted microscope to observe the scratch width changes. The migration rate was calculated using the following formula: (scratch distance at 0 h–scratch distance at 24 h)/scratch distance at 0 h. Three replicates were used for each experiment.

### 2.6. Cell Migration Assay

Cell invasion ability was determined using a transwell chamber (Corning, NY, USA). The 2,000 stably transfected cells and the cells in the HCs were suspended in medium containing 1% FBS and then seeded onto the upper chamber. The insert was coated in advance with Matrigel at 37°C for 2 h. Next, the medium (0.8 mL medium supplemented with 10% FBS) was added to the lower chamber. Twenty-four hours later, the cells on the surface of the upper membrane were removed with a cotton swab, and the cells that passed through the insert were fixed with 4% paraformaldehyde for 30 min, stained with 10% Giemsa staining solution, and washed with PBS thrice. The number of invading cells was counted using an inverted microscope. Three replicates were used for each experiment.

### 2.7. Annexin V-Fluorescein Isothiocyanate (Annexin V-FITC) Staining

The cells were harvested and counted after reaching 80% confluence. The cells (1 × 10^6^) were rinsed with precooled PBS twice, suspended in 1 × Annexin buffer, and incubated for 10 min with 5 *μ*L AnnexinV-FITC (Becton Dickinson, Franklin Lakes, NJ, USA) without light exposure. Subsequently, the cells were rinsed once with precooled PBS and suspended in 300 *μ*L of 1 × Annexin buffer. A flow cytometer (Guava EasyCyte HT; Millipore, Billerica, MA, USA) was used to detect the cell apoptosis rate.

### 2.8. RNA Pull-Down Assay

Depending on the manufacturer's instructions, the RNA drop-down assay was conducted using the Pierce™ magnetic RNA protein pull-up kit (Thermo Fisher Scientific, Waltham, MA, USA). Biotin-labeled lncRNA FAM225B, antisense lncRNA FAM225B, and control RNA probes were synthesized using base factors (https://www.genepharma.com). In brief, the labeled RNA probe was bonded to streptavidin magnetic beads and incubated with cleaved SKOV3 cells. The RNA-binding protein complex was eluted and separated using sodium dodecyl sulfate-polyacrylamide gel electrophoresis. The expression of DDX17 in the drop-down products was tested using Western blotting.

### 2.9. Radioimmunoprecipitation (RIP) Assay

RIP analysis was performed using the Millipore EZ-Magna RIP RNA-binding protein immunoprecipitation kit (Millipore). SKOV3 cells were collected and dissolved in an RNase-free radioimmunoprecipitation assay buffer (Beyotime, Shanghai, China). The protein A/G beads were pre-extracted and then incubated with DDX17 antibodies (1 : 100, PA5-84585, Invitrogen, New York, USA). Subsequently, the protein A/G beads were incubated overnight with the supernatant. The total RNA was extracted from the RIP product, and the enrichment of lncRNA FAM225B in the complex was determined using reverse transcription-quantitative polymerase chain reaction (RT-qPCR).

### 2.10. Chromatin Immunoprecipitation (ChIP) Assay

The binding of DDX17 and PDIA4 promoters was analyzed using a ChIP kit (Millipore). Formaldehyde was used to crosslink DNA and proteins, and the samples were fixed for 30 min. The DNA fragments separated from SKOV3 cells were further fragmented into 2,001,000 bp using ultrasonic treatment. After incubating the fragmented DNA with DDX17 (1 : 100, PA5-84585, Invitrogen) or an IgG antibody, the settling DNA fragment was determined using RT-qPCR. The primer sequences used were as follows: F: 5′-GGCGTCAGTCTGGGATTGG-3′ and R: 5′-TTTCCGGGGCCTCATGGTAG-3′.

### 2.11. RT-qPCR

The cells were first dissolved in 1 mL TRIzol (Thermo Fisher Scientific), and the total RNA was extracted according to the manufacturer's specifications. The total RNA was reverse transcribed into cDNA using M-MLV reverse transcriptase (D7160L, Beyotime) and random primers. The reaction system was configured according to the Premix EX Taq™ II Kit (Takara, Dalian, China), following the manufacturer's instructions. An ABI7500 quantitative PCR (Applied Biosystems, Shanghai, China) was used for RT-qPCR with glyceraldehyde phosphate dehydrogenase (GAPDH) as a loading control for lncRNA and mRNA. The relative expression level was determined by the 2^−ΔΔCt^ method [22]. The primers used are listed in Table 1.

### 2.12. Western Blot Assay

The cells were lysed with lysis buffer, and the total protein concentration was determined using a bicinchoninic acid protein detection kit (23227, Thermo Fisher Scientific). The protein samples were then diluted with 5 × sample buffer. For detection of protein in the serum samples, serum protein concentration was measured and diluted to 5 *μ*g/uL. Then, the appropriate volume of loading buffer was added, and the mixture was boiled at 100°C for 5 min. Albumin/IgG was removed using the Albumin/IgG Depletion Kit (37591; Qiagen, Germany) according to the manufacturer's instructions. Proteins were separated for 90 min in a 12% separation gel and incubated for 1 h in a PBS blocking solution containing 5% (w/v) evaporated skimmed milk. Next, the cells were incubated with the primary antibodies against DDX17 (1 : 300, PA5-84585, Invitrogen), PDIA4 (1 : 1000, AB155800, Abcam, Cambridge, UK), and *β*-actin (1 : 5000, AB8277, Abcam) at 4°C, overnight. The membrane was then rinsed and incubated for 1 h with the secondary antibody (1 : 5000, ab114610, Abcam). Finally, proteins were detected using an enhanced chemiluminescence method, and images were captured using a Bio-Spectrum Gel Imaging System (UVP, Upland, CA, USA).

### 2.13. Statistical Analysis

All experiments were repeated thrice unless otherwise stated. SPSS 18.0 (IBM Corp., Armonk, NY, USA) and GraphPad Prism 8.0 (GraphPad Software Inc.) were used for statistical analysis. All data are shown as the mean ± standard deviation. Two groups of data were evaluated using a *t* test. Multiple groups of data were assessed using one-way analysis of variance (ANOVA). Tukey's post hoc test was used for post hoc multiple comparisons. *P* significance was set at *P* < 0.05.

## 3. Results

### 3.1. Reduced Expression of lncRNA FAM225B and PDIA4 Observed in Patients with Ovarian Cancer

**Figure 1:**
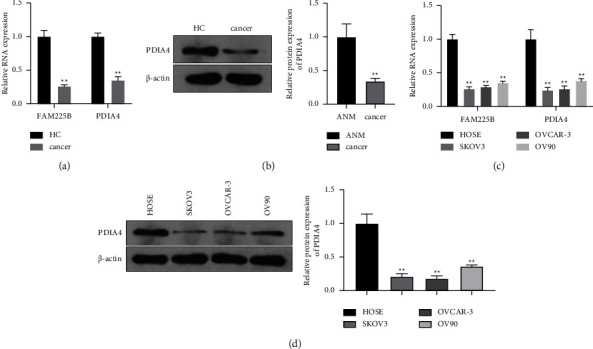
Low expression levels of lncRNA FAM225B and PDIA4 are found in ovarian cancer. RT-qPCR (a) and Western blot assay (b) were carried out to detect the expression levels of lncRNA FAM225B and PDIA4 in the collected serum of ovarian cancer patients and healthy controls. RT-qPCR (c) and Western blot assay (d) were carried out to detect the expression levels of lncRNA FAM225B and PDIA4 in ovarian cancer cells. *N* = 3; ^*∗*^*P* < 0.05; ^*∗∗*^*P* < 0.01.

First, lncRNA FAM225B and PDIA4 expression levels in the serum of patients with ovarian cancer (cancer group) and HCs were determined using RT-qPCR and Western blot assays. These findings demonstrated that the lncRNAs FAM225B and PDIA4 were downregulated in the serum of patients with ovarian cancer compared to the serum in HCs (Figures 1(a) and 1(b); *P* < 0.01). To further verify the differential expression of FAM225B and PDIA4 in ovarian cancer cells, we tested their expression levels in ovarian cancer cell lines. It was hypothesized that the lncRNAs FAM225B and PDIA4 expression were reduced in ovarian cancer cells compared with HOSEPIC cells (Figures 1(c) and 1(d); *P* < 0.01). This suggests that FAM225B and PDIA4 are involved in ovarian cancer.

### 3.2. Ovarian Cancer Cell Invasion and Migration Are Inhibited by LncRNA FAM225B

**Figure 2:**
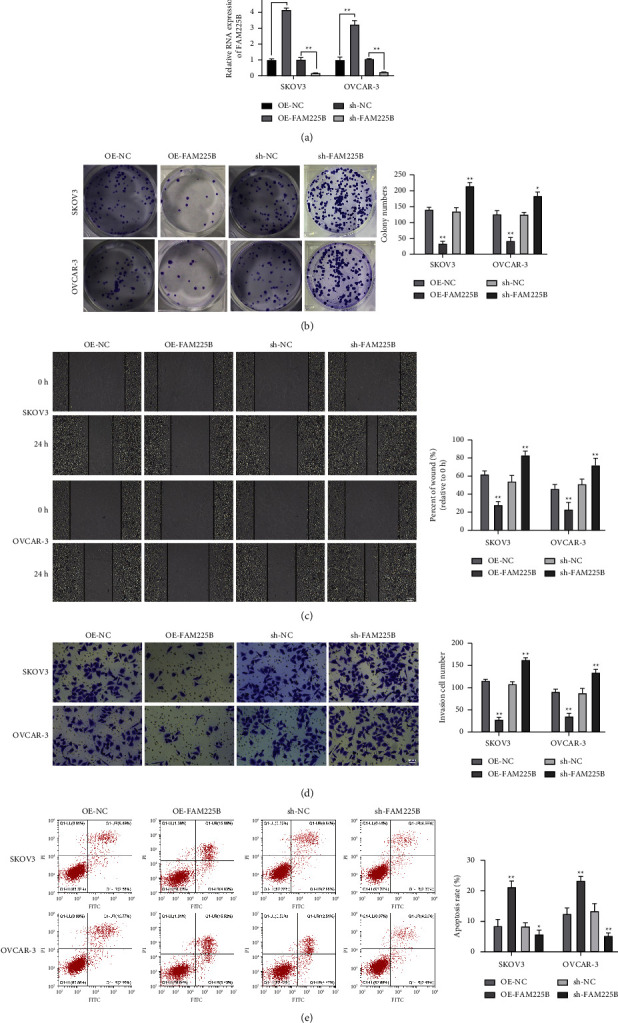
Overexpression of lncRNA FAM225B suppresses the ovarian cancer cell progression. LncRNA FAM225B-associated vectors were transfected in SKOV3 and OVCAR-3 cells: the transfection efficiency was verified by RT-qPCR (a). Colony formation assay (b), wound healing assay (c), cell migration assay (d), and flow cytometry (e) were performed to detect ovarian cancer cell proliferation ability, migration ability, invasion ability, and cell apoptosis rate, respectively. *N* = 3; ^*∗∗*^*P* < 0.01.

On the basis of the levels of lncRNAs FAM225B and PDIA4 in tumor cell lines, we chose the SKOV3 and OVCAR-3 cell lines for further research. To elucidate the effect of lncRNA FAM225B on the development of ovarian cancer, we transfected both the FAM225B overexpression- and silenced expression-related vectors into SKOV3 and OVCAR-3 cells. The transfection efficiency was tested using RT-qPCR. The corresponding results revealed that SKOV3 and OVCAR-3 cells treated with OE-FAM225B exhibited increased lncRNA FAM225B expression levels, and the level was decreased in cells treated with sh-FAM225B (Figure 2(a), both *P* < 0.01), indicating that the vector was successfully transfected.

Subsequently, colony formation, wound healing, cell migration, and flow cytometry assays were performed to detect ovarian cancer cells' ability to proliferate, migrate, invade, and undergo apoptosis, respectively. The results suggest that treatment with OE-FAM225B decreased the ability of SKOV3 and OVCAR-3 cells to proliferate (Figure 2(b); *P* < 0.01), migrate (Figure 2(c); *P* < 0.01), and invade (Figure 2(d); *P* < 0.01) and increased cell apoptosis (Figure 2(e); *P* < 0.01). In contrast, SKOV3 and OVCAR-3 cells treated with sh-FAM225B exhibited increased ability to proliferate, migrate, and invade and decreased apoptosis rates (Figures 2(b)–2(e); all *P* < 0.01). These results imply that lncRNA FAM225B suppresses ovarian cancer cell growth and promotes apoptosis.

### 3.3. PDIA4 Restricts Ovarian Cancer Cell Invasion and Migration

**Figure 3:**
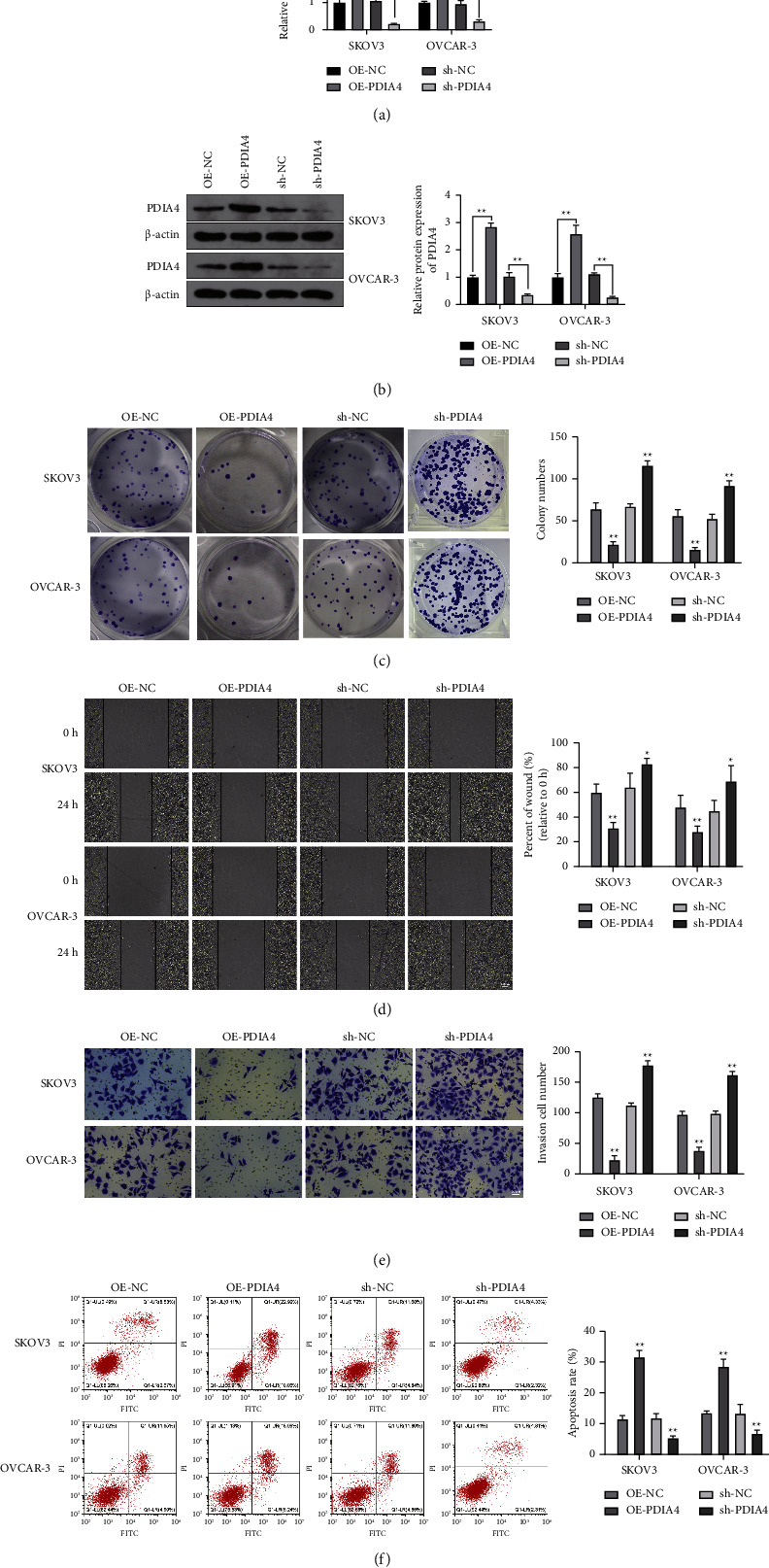
Upregulation of PDIA4 inhibits the ovarian cancer cell progression. PDIA4-associated vectors were transfected in SKOV3 and OVCAR-3 cells: the transfection efficiency was verified by (a) RT-qPCR and (b) Western blot assay. Colony formation assay (c), wound healing assay (d), cell migration assay (e), and flow cytometry (f) were performed to detect ovarian cancer cell proliferation ability, migration ability, invasion ability, and cell apoptosis rate, respectively. *N* = 3; ^*∗*^*P* < 0.05; ^*∗∗*^*P* < 0.01.

To determine the effect of PDIA4 on ovarian cancer cells, we transfected SKOV3 and OVCAR-3 cells with PDIA4-related vectors. The results of the RT-qPCR and Western blot assays suggested a higher expression level of PDIA4 in SKOV3 and OVCAR-3 cells transfected with OE-PDIA4, whereas the expression levels decreased in cells treated with sh-PDIA4 (Figures 3(a) and 3(b); both *P* < 0.01), demonstrating that the vectors were successfully transfected.

Next, the phenotype of ovarian cancer cells was examined. The corresponding findings demonstrated that OE-PDIA4 transfected SKOV3 and OVCAR-3 cells exhibited suppressed proliferation, migration, and invasion abilities and an elevated apoptosis rate (Figures 3(c)–3(f); all *P* < 0.01). In contrast, increased proliferation, migration, and invasion abilities and a decreased apoptosis rate were observed in sh-PDIA4 transfected SKOV3 and OVCAR-3 cells (Figures 3(c)–3(f); all *P* < 0.01). These results indicate that PDIA4 acts as a tumor suppressor in ovarian cancer.

### 3.4. LncRNA FAM225B Promotes PDIA4 Expression by Binding to Transcription Factor DDX17

**Figure 4:**
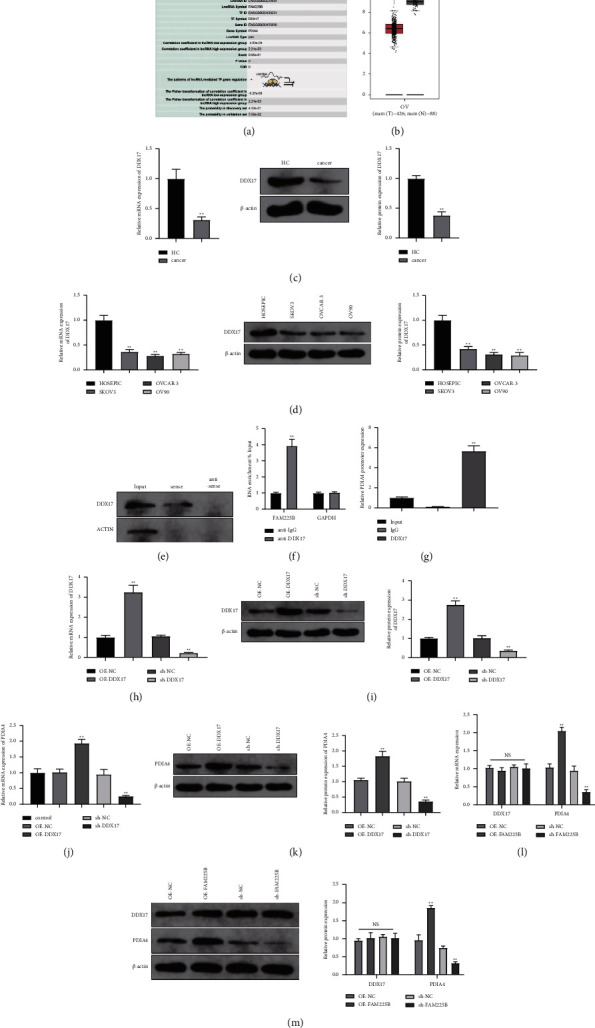
LncRNA FAM225B positively regulates PDIA4. Detection of the interaction between lncRNA FAM225B and PDIA4: (a) LncMAP predicted the role of lncRNA FAM225B and PDIA4 in ovarian cancer. (b) GEPIA database queried the expression of DDX17 in ovarian cancer. RT-qPCR and Western blot assay were carried out to detect the expression levels of DDX17 mRNA and protein in serum (c) and cell lines (d) of ovarian cancer. RNA pull-down assay (e) and RIP assay (f) were performed to detect the binding between lncRNA FAM225B and DDX17. (g) ChIP assay was used to detect the binding of DDX17 and PDIA4 promoter. RT-qPCR (h) and Western blot assay (i) were carried out to determine the transfection efficiency of DDX17-related vectors. (i–m) RT-qPCR and Western blot assay were implemented to detect the expression of PDIA4 after cell tranasfection. *N* = 3, ^*∗∗*^*P* < 0.01.

To determine whether PDIA4 participates in the molecular mechanism of lncRNA FAM225B in the development of ovarian cancer, we predicted that lncRNA FAM225B would inhibit PDIA4 expression by binding to the transcription factor DDX17 (Figure 4(a)). The GEPIA database showed that DDX17 was lowly expressed in ovarian cancer (Figure 4(b)). The RT-qPCR and Western blot assays revealed that DDX17 was expressed at low levels in the serum of patients and cell lines of ovarian cancer (Figures 4(c) and 4(d); both *P* < 0.01). Subsequently, an RNA pull-down assay was conducted to collect the protein bound to lncRNA FAM225B in SKOV3 cells, and a Western blot assay was conducted to test DDX17 expression in the samples. The results showed that DDX17 could directly bind to lncRNA FAM225B (Figure 4(e)). The RIP results indicated that lncRNA FAM225B could directly interact with DDX17 in SKOV3 cells (Figure 4(f); *P* < 0.01). Furthermore, ChIP analysis revealed that the PDIA4 promoter was enriched in the complex pulled down by the DDX17 antibody (Figure 4(g)), indicating that DDX17 could bind to the PDIA4 promoter.

We used the RT-qPCR and Western blot assays to detect transfection deficiency in SKOV3 cells after transfecting the DDX17-related vector (Figures 4(h) and 4(i); *P* < 0.01). PDIA4 expression was tested after successful transfection. The results suggested that SKOV3 and OVCAR-3 cells treated with OE-DDX17 exhibited a higher PDIA4 expression level, whereas sh-DDX17-treated SKOV3 and OVCAR-3 cells showed reduced PDIA4 expression levels (Figures 4(j) and 4(k); both *P* < 0.01). However, the change in DDX17 expression was not significant after FAM225B differential expression. A higher expression level of PDIA4 was found in cells treated with OE-FAM22B, and a lower expression level of PDIA4 was observed in cells transfected with sh-FAM225B (Figures 4(l) and 4(m); *P* < 0.01). These findings reveal that the lncRNA FAM225B has no regulatory effect on DDX17 expression but may promote PDIA4 expression when combined with DDX17.

### 3.5. LncRNA FAM225B Inhibits Ovarian Cancer Cell Progression via Upregulating PDIA4

**Figure 5:**
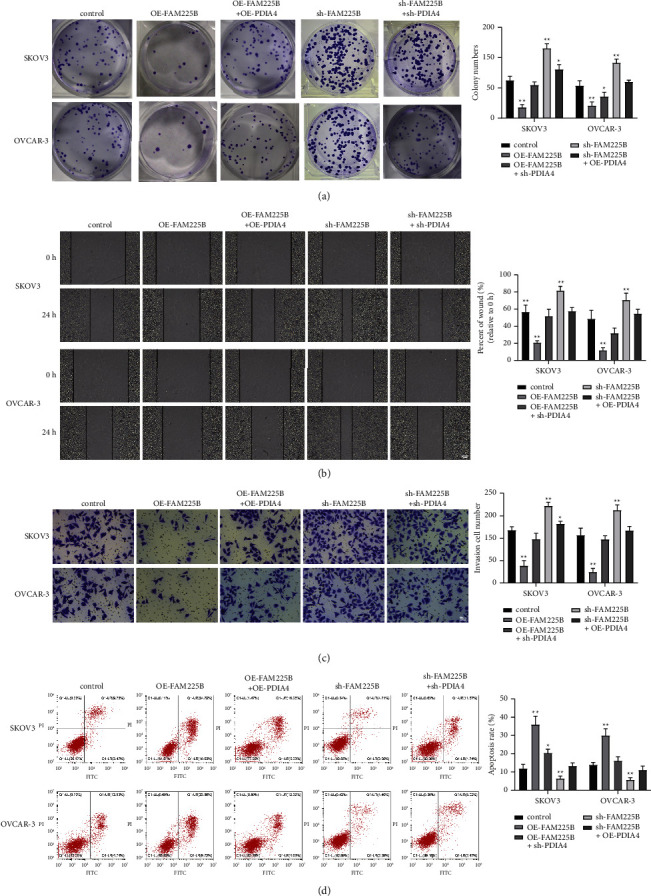
LncRNA FAM225B inhibits ovarian cancer cell growth by upregulating PDIA4. Transfection or cotransfection of lncRNA FAM225B- and PDIA4-related vectors in ovarian cancer cells: colony formation assay (a), wound healing assay (b), cell migration assay (c), and flow cytometry (d) were performed to detect ovarian cancer cell proliferation ability, migration ability, invasion ability, and cell apoptosis rate, respectively. *N* = 3, ^*∗*^*P* < 0.05, ^*∗∗*^*P* < 0.01.

To determine if FAM225B regulates PDIA4 expression in ovarian cancer, we tested the phenotypes of the cells after transfection or cotransfection with lncRNA FAM225B- and PDIA4-related vectors. The results revealed that in SKOV3 and OVCAR-3 cells, the downregulation of PDIA4 reversed the inhibitory effect of the upregulated lncRNA FAM225B. In addition, the upregulation of PDIA4 in SKOV3 and OVCAR-3 cells neutralized the stimulatory effect of the downregulated lncRNA FAM225B (Figures 5(a)–5(d); all *P* < 0.01). The results suggest that the lncRNA FAM225B can increase PDIA4 expression by combining with DDX17, inhibiting the process of ovarian cancer.

## 4. Discussion

Ovarian cancer is a lethal malignancy with a poor prognosis. Therapeutic strategies for this disease involve surgery and chemotherapy [23]. In addition, suitable biomarkers have been identified to facilitate early diagnosis, prognostic assessment, and the evaluation of treatment response [3, 24]. Despite efforts to prevent this disease, ovarian cancer poses a significant threat, especially for individuals without the typical symptoms in the early stages [25]. Therefore, the timely detection and effective treatment of this disease remain top priorities for researchers in the field of ovarian cancer. In this study, we demonstrated that lncRNA FAM225B elevates PDIA4 by directly binding to DDX17, restricting the biological function of ovarian cancer cells.

Abnormal expression or mutations in lncRNAs have been reported to correlate with ovarian cancer development at different stages [26]. In addition, lncRNA researchers have proposed novel approaches for the diagnosis and prognosis of ovarian cancer at the intracellular and exosomal lncRNA levels [27]. We observed a low expression level of lncRNA FAM225B in ovarian cancer that lncRNA FAM225B overexpression suppressed ovarian cancer progression and that lncRNA FAM225B suppression exhibited an inverse trend. According to the previous studies, lncRNA FAM225B expression is dramatically elevated in nasopharyngeal carcinoma and recurrent glioblastoma, and lncRNA FAM225B knockdown restricts cell proliferation, migration, and invasion [28, 29]. Thus, we inferred that lncRNA FAM225B might have a triggering or suppressive effect on the biological processes of various cancer forms. Similarly, other lncRNAs have been found to play an inhibitory role in ovarian cancer. For instance, a report suggested that lncRNA GAS5 is a tumor suppressor in ovarian cancer, with decreased expression in cancer cells [30]. Wang et al. reported that lncRNA MEG3 is downregulated in ovarian cancer, and overexpression of MEG3 induces apoptosis and prevents invasion and migration [31].

PDIs are a family of proteins with many functions, including chaperone activity, redox reactions, and protein folding [32]. Several PDI isoforms (PDIA1, 3, and 6) are modulated by estrogen in cancer cells [33]. Especially, downregulated PDIA4 has been observed in patients with platinum-resistant ovarian cancer [34]. Similarly, in our study, the downregulation of PDIA4 was observed in ovarian cancer, and the restoration/downregulation of PDIA4 contributed to the restriction/promotion of ovarian cancer cell progression. Consistently, Chanjiao et al. revealed that PDIA4 expression is downregulated in ovarian cancer and that overexpression of PDIA4 tends to suppress the malignant phenotypes of ovarian cancer cells [35]. Because of the involvement of PDIA4 in various diseases, a comprehensive understanding of its potential clinical value is urgently required.

Furthermore, to elucidate the molecular mechanism of action of lncRNA FAM225B in the development of ovarian cancer, we verified the direct interaction between lncRNA FAM225B and transcription factor DDX17 in ovarian cancer cells using RNA pull-down and RIP assays. Li et al. supported a strong association between lncRNA FAM225B and transcription, and that lncRNA FAM225B mainly plays a role in the transcription and regulation of transcription [29]. The ChIP assay suggested that the PDIA4 promoter was also enriched in the complex pulled down by the DDX17 antibody. DDX17 performs vital cellular functions, including splice site selection, RNA splicing, and rearrangement of secondary RNA structures [36, 37]. Emerging evidence has indicated that DDX17 acts as a transcriptional coregulator [38] or a cofactor of microprocessors in cancer development, such as in non-small cell lung cancer, glioma cells, and hepatocellular carcinoma [39–41]. However, the interactions among these three factors in ovarian cancer require further investigation.

In conclusion, the main findings of our study indicate that lncRNA FAM225B plays an inhibitory role in ovarian cancer via the DDX17/PDIA4 axis. The use of lncRNA FAM225B is highly promising in diagnosis and prognosis. In addition, the rapidly increasing review of lncRNA biology will provide valuable applications to improve the treatment of ovarian cancer.

## Figures and Tables

**Table 1:** Primer sequences in this study.

Name of primer	Sequences (5′-3′)
FAM225B-F	TTCCCGGCTCTCCTTAGACA
FAM225B-R	TCTTTTCCAGCGGTCACTCC
DDX17-F	TGTCAGCCTTGCTACTTCCG
DDX17-R	GGCATGTACGATACGTTGCT
PDIA4-F	CCTGCAGAAATTAGAACGCGG
PDIA4-R	CCACCAGCTTTGTAACCAGTC
GAPDH-F	ACCACAGTCCATGCCATCAC
GAPDH-R	TCCACCACCCTGTTGCTGTA

*Note.* F, forward primer; R, reverse primer; GAPDH, glyceraldehyde phosphate dehydrogenase.

## Data Availability

All data are accessible upon reasonable request from the corresponding authors.
